# Physical Activity Patterns and Behavioral Resilience Among Foggia University Students During the COVID-19 Pandemic: A Public Health Perspective

**DOI:** 10.3390/healthcare14010087

**Published:** 2025-12-30

**Authors:** Tarek Benameur, Neji Saidi, Maria Antonietta Panaro, Chiara Porro

**Affiliations:** 1 Department of Biomedical Sciences, College of Medicine, King Faisal University, Al Ahsa 31982, Saudi Arabia; 2 Department of Mathematics and Statistics, College of Sciences, King Faisal University, Al Ahsa 31982, Saudi Arabia; 3 Department of Biosciences, Biotechnologies and Environment, University of Bari, I-70125 Bari, Italy; 4 Department of Clinical and Experimental Medicine, University of Foggia, I-71100 Foggia, Italy

**Keywords:** physically active lifestyle, physical activity, students, lockdown, exercise, health, lifestyle, gender differences, COVID-19, pandemic, resilience

## Abstract

**Background**: The (COVID-19) pandemic profoundly disrupted daily routines and physical activity (PA), especially among university students, due to restrictions and limited access to sports facilities. As this group is particularly vulnerable to sedentary lifestyles and mental health issues, understanding their PA patterns is crucial. This study explores overall and domain-specific PA levels and the influence of sociodemographic factors, offering insights for promoting sustainable PA strategies in higher education during and beyond health crises. **Methods**: A cross-sectional online survey was conducted among University of Foggia students during the pandemic. The participants completed the validated Italian IPAQ-Long to assess PA across various domains. Associations with demographics and perceived barriers were analyzed via *t* tests, ANOVA, and nonparametric tests. **Results**: A total of 301 students completed the survey. Despite barriers such as limited living space, low income, and sports facility closures, 66% of the participants reported high PA levels, mainly through work-related and leisure activities. This remains insufficient. PA varied significantly by gender, income, residence, and employment status: males reported higher leisure PA, whereas females engaged more in active transport and domestic activities. Rural residents and those with moderate incomes demonstrated higher overall PA, whereas employed students presented lower activity levels. These findings underscore the complex socioeconomic and environmental factors shaping PA behavior during an unprecedented global health crisis. **Conclusions**: The findings reveal that students’ resilience in maintaining PA is a coping mechanism despite socioeconomic and environmental barriers. Tailored, accessible PA initiatives integrated into university curricula can enhance student well-being, academic performance, and long-term health during and after public health emergencies. Universities should adopt accessible, equity-oriented PA initiatives to promote physical and mental health and enhance public-health preparedness during future emergencies.

## 1. Introduction

Severe acute respiratory syndrome coronavirus 2 (SARS-CoV-2), which is responsible for coronavirus disease 2019 (COVID-19), was declared a pandemic on 11 March 2020. This pandemic has led to a worldwide global health crisis [1]. Its impact is massive in various life domains. To curb the rapid spread of coronavirus disease 2019 (COVID-19), governments have adopted rigorous public health strategies. This has entailed the implementation of strict lockdowns, the enforcement of social and physical distancing, and the introduction of restrictive policy measures, by which population movement has been heavily limited and restricted [2].

Towards the end of February 2020, Italy emerged as the epicenter of the COVID-19 pandemic in Europe, prompting the implementation of nationwide quarantine measures on 11 March 2020 [3]. These measures included stringent restrictions on movement outside of the house, except for proven needs or individual exercise within the limited vicinity of one’s home. Furthermore, in response to the challenges posed by the SARS-CoV-2 crisis, both commercial enterprises and academic institutions were compelled for full-time remote work transitions. This has led to a comprehensive shift in all educational activities to online classes.

The imposed lockdown resulted in the temporary suspension of various business activities, gathering in public places, fitness and physical activity (PA) centers, fewer opportunities to exercise with others, and overall negative effects on social life. This has affected many aspects of not only the general population but also students’ lives, including their fitness activities, which has resulted in various psychological disorders (e.g., stress, anxiety, depression), serious fitness, and health concerns [4,5]. During the period of the emergence of new variants, a resurgence of restrictive measures ensued, resulting in the closure of sports facilities, including gym, sports and fitness centers. The Italian government has urged these establishments to encourage “remote training” where possible, offering exercise classes and online consultations as feasible substitutes for in-person engagement. Moreover, emphasis was placed on the promotion of home-based exercise, thereby fostering a culture of preserving health and well-being within domestic confinement.

Therefore, great concerns have been raised about the risk of potential adverse impacts on both physical and mental health, especially among quarantined people, especially among university students, who have been particularly affected by the COVID-19 pandemic [6,7,8,9]. Such circumstances imply that satisfying minimal levels of PA necessary for sustaining health would have been compromised [10].

Furthermore, university students are highly susceptible to weight gain and early onset of chronic diseases, including hypertension, dyslipidemia, and cardiovascular disorders driven by sedentary lifestyles, limited PA, limited PA, and instances of binge drinking [11,12,13,14]. Italian university students have shown low levels of mental well-being and the highest rates of anxiety, depression, and suicidal risk, further amplifying these lifestyle-related health risks [15]. These changes contributed to unhealthy eating patterns, weight gain, elevated BMI, and prolonged sedentary behavior, with many behaviors persisting post-pandemic and showing sex-specific differences [16]. The combination of reduced PA, poor sleep, and heightened stress, create an emerging risk profile for chronic conditions such as hypertension and type 2 diabetes, as well as cardiometabolic disorders including dyslipidemia, insulin resistance, and cardiovascular disease, obesity, which may develop early when unhealthy behaviors are adopted during young adulthood [17].

In addition, PA has an impact on students’ cognitive skills, such as concentration and attention, and enhances class attitudes and behaviors, all of which are important components of improved academic performance [18]. PA not only is helpful in managing the psychological stress commonly encountered by students but also has a positive impact on mood, self-esteem, physical and mental well-being, overall quality of life, and the prevention of chronic health conditions [19,20,21,22].

On the basis of World Health Organization (WHO) guidelines and the American College of Sports Medicine (ACSM), performing at least 150 min of moderate-intensity (or 75 min of vigorous-intensity) aerobic PA per week, together with two or more days per week for muscle-strengthening activities, is recommended. It is also recommended to limit sedentary behavior throughout the day [23].

During the pandemic, various regions and autonomous provinces in Italy were classified into four categories: red, orange, yellow and white. These classifications indicate different risk levels, each corresponding to specific restrictive measures aimed at controlling the spread of the virus. Foggia, which constitutes the focus of our study area, has been categorized into the orange zone, indicating a moderate level of risk. The impacts of lockdowns and restrictive health measures on PA levels and sedentary behavior among university students in Italy remain unclear, especially during the emergence of various SARS-CoV-2 variants. This highlights the urgent need to determine how to intervene and reverse possible detrimental effects on physical and mental health.

Furthermore, during the various waves of COVID-19 infections in Italy, PA continued to decline. Although activity levels improved as restrictions gradually eased, they frequently remained below prepandemic baselines. Furthermore, emerging evidence suggests that beyond physical inactivity, university young students infected with COVID-19 may also experience persistent cognitive impairments such as reduced attention, memory deficits, and mental fatigue.

These sequelae have been observed up to four years post-infection and are believed to be exacerbated by the timing of infection relative to variant surges and vaccination status, raising concerns about their long-term academic and occupational implications [24]. Alarmingly, mental health challenges were not limited to the Italian general population as previously described [25]. University students were particularly affected, exhibiting the lowest levels of well-being and the highest rates of anxiety, depression, and suicidal risk during the pandemic.

Hence, our study aimed to explore the impact of the COVID-19 pandemic on PA patterns and levels among the university student population at Foggia University and the factors that predict PA changes.

## 2. Materials and Methods

### 2.1. Study Design and Settings

A cross-sectional survey was designed using a validated, anonymous, self-administered questionnaire [26]. The study involved a convenience sample of university students from various academic disciplines and education levels. The survey was conducted in Italy between July 2021 and March 2022 during the COVID-19 pandemic period characterized by the emergence and circulation of successive SARS-CoV-2 variants [27]. This timeframe includes the delta and Omicron waves, during which public health measures, vaccination campaigns, and varying degrees of restrictions were still in place.

Although some normalization efforts had begun, the period was still characterized by significant pandemic-related influences on public life and behavior.

### 2.2. Participants

Participants were recruited using convenience sampling, a non-probability approach comprising a sample of 301 students from various specialties and educational levels. The majority were enrolled in Sciences of Motor and Sports Activities, followed by Nursing Sciences, Radiology Technology, Medicine and Surgery, Dentistry, and Biomolecular Sciences and Technologies. Smaller groups were from Psychological and Education Sciences, Business and Economics, Humanities and Arts, Technical/Scientific High Schools.

The participants were invited to complete an online survey during the COVID-19 pandemic. The survey link was distributed through university communication channels, including institutional mailing lists and student networks, to ensure broad representation across different disciplines and academic stages.

Participation was entirely voluntary, and the students were encouraged to take part without any undue pressure. Informed consent was obtained from all participants prior to survey completion. Additionally, participants were also informed of their right to withdraw from the study at any point without any consequences.

#### 2.2.1. Inclusion Criteria for the Participants

The participants meeting the following criteria were eligible for the study:

Aged 18 years or older and enrolled at the University of Foggia during the academic year in which the study was conducted.

The patient reported no prior history of physical and/or psychological disorders.

#### 2.2.2. Exclusion Criteria for the Participants

Participants were excluded from the study if they met any of the following criteria: The patients reported a diagnosis of any physical and/or psychological condition or were currently receiving any kind of drug treatment.

Had musculoskeletal diseases or injuries that impaired mobility and potentially prevented active participation in the study or interfered with the completion of the pretesting procedures.

### 2.3. Study Instrument

The online survey was developed from the International Physical Activity Questionnaire (IPAQ) to measure the level of PA among university students during the COVID-19 pandemic. The PA level was assessed through the Italian validated long version of the IPAQ, which has been culturally adapted and validated for use in the Italian population as previously describe [26,28,29,30]. The original English version of the questionnaire was translated into Italian, according to the standardized procedures by two independent bilingual experts and was subsequently pretested for content validity, readability, comprehension and design in a pilot study conducted on randomly selected students from the survey’s intended population.

The online survey also collected sociodemographic and anthropometric data, including age, sex, educational level, monthly income, academic specialty, marital status, preferred sports habits, place of residence, reported obstacles to PA, weight, height and employment status. The IPAQ provides a quantitative score (in METs-min/week) for PA performed in the seven days prior to questionnaire completion across five domains (job-related PA (7 items); transportation PA (6 items); housework, house maintenance and family care (6 items); recreational sport and leisure-time PA (6 items); and sitting time (2 items)).

The 27-item IPAQ (long form) is divided into five sections: work, transportation, domestic and recreational activities, and sitting time. The first section assesses PA related to professional work, agriculture, and other social work outside the home. The second section evaluates the time spent traveling for work, shopping, entertainment, whether by walking, or using transport modes such as car, bicycle, and public transport. The third section focuses on the time spent on housework cleaning and family care. The fourth section estimates the time of PA dedicated to recreational, sport, or leisure activities. The final section contains questions about the time spent sitting during work, leisure, and study, such as sitting at a desk, watching television, or reading. The questionnaire considers activities performed during the last 7 days, with each session lasting at least 10 min. In accordance with the IPAQ guidelines and considering the frequency, intensity, duration, and type of PA performed, participants were classified into three physical activity levels: “low PA”, “moderate PA” and “high PA”.

The online survey was conducted via the Google Forms web survey platform (Google LLC, Mountain View, CA, United States) and was communicated via the official channels of the University of Foggia and/or through e-mails if needed.

**Figure 1:**
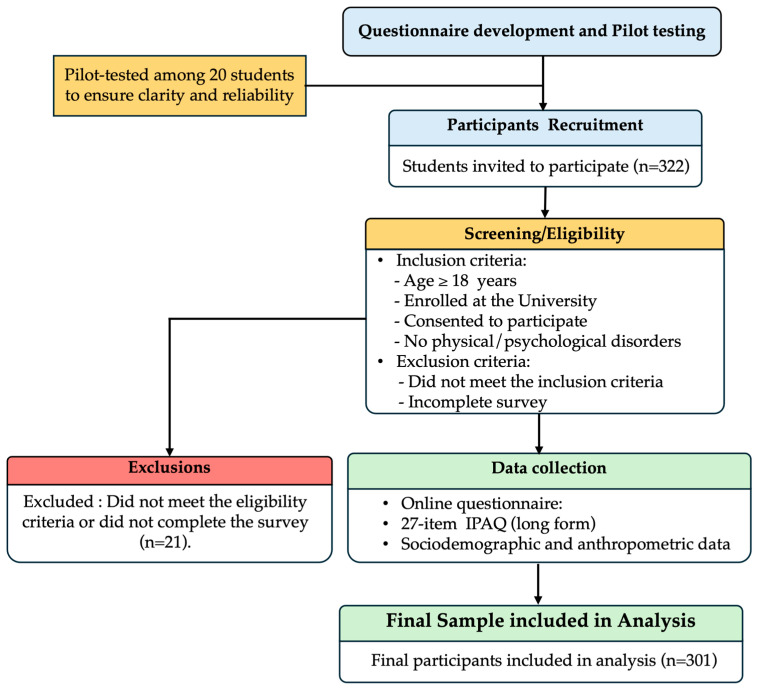
Flow diagram of the study.

As shown in Figure 1, the flow diagram clearly depicts the study design, pilot testing of the questionnaire, and participant flow through each step of the survey including the inclusion, exclusion criteria and the number of total participants included in the study analysis.

### 2.4. Outcome Measure and Data Analysis

On the basis of the protocol developed by the International IPAQ Committee [26,28] the physical activity (PA) levels were converted into metabolic equivalents (METs), where 1 MET corresponds to the resting metabolic rate, defined as 3.5 mL O_2_ kg^−1^ min^−1^ or 1 kcal kg^−1^ h^−1^. The weekly PA level was expressed as energy expenditure in MET-minutes per week (MET-min/week), which was calculated by multiplying the MET coefficient assigned to each activity by the number of days per week the activity was performed and its average duration in minutes. The MET coefficients used were 8.0 for vigorous-intensity physical activities, 6.0 for cycling, 4.0 for moderate-intensity physical activities, and 3.3 for walking. These scores are used to classify individuals into categories of low, moderate, or high PA levels. This classification helps assess whether respondents meet the recommended levels of PA for health benefits. The total PA per week was calculated by summing the MET-min/week values for all reported walking, moderate-intensity, and vigorous-intensity activities [27]. Additionally, total sedentary behavior was estimated via the following formula: weekday sitting time ×5 + weekend sitting time ×2 + sitting time during transport. On the basis of the collected data, participants were categorized into three PA levels according to the classification criteria provided in the IPAQ protocol.

### 2.5. Statistical Analysis

A descriptive and comparative statistical data analysis was performed using the SPSS software package, version 26.0 (IBM Corp., Armonk, NY, USA), and the means and standard deviations (SDs) were calculated for continuous variables such as age. The level of statistical significance was set at α = 0.05. The normality of the data distribution was assessed via the Shapiro-Wilk test. Continuous variables are presented as the mean ± standard deviation (SD). For normally distributed continuous variables, independent samples *t* tests were used to compare means between two groups, and one-way analysis of variance (ANOVA) was used for comparisons across more than two groups. To compare the differences in the level of PA, Kruskal–Wallis one-way analysis of variance was used. Continuous variables are presented as the means ± SDs.

## 3. Results

### 3.1. Sociodemographic Characteristics of the Study Population

The sociodemographic characteristics of the respondents are presented in Table 1. A total of 301 students completed the online IPAQ (long form). The mean age of the participants was 22.03 ± 4.9 years (SD), with a median of 21 years and a range of 18–49 years, indicating that a relatively young population sampled for the study. The age categories revealed a predominant presence of individuals aged 18–20 years (49.2%) and 21–30 years (45.2%). Among the surveyed students, 132 were male, which represents 43.9% of the sample, and 169 were female, accounting for 56.1% of the total respondents. In terms of educational background, most of the respondents were undergraduates, comprising 272 individuals, making up 90% of the sample.

When considering living status during the pandemic, most of the respondents 285 (94.7%) reported living with their families/others. Regarding monthly income, the majority of respondents reported earnings below 500 euros, constituting 64.1% of the sample, followed by 21.9% of respondents with monthly incomes above 1000 euros. This highlights that a significant portion of the respondents had relatively low monthly incomes.

With respect to BMI distribution, the majority of the respondents fell within the normal weight category (18.5–24.9), comprising 222 (73.8%) of the sample, followed by 46 (15.3%) classified as preobese, whereas only a small fraction fell into the underweight (9.3%) or obese categories 5 (1.7%).

The following questions are related to the living conditions and PA patterns of the respondents during the COVID-19 pandemic. It is obvious that the respondents were living in diverse living spaces, with nearly half of them (45.8%) living in spaces ranging from 10 to 100 m^2^, followed by 41.5% living in spaces ranging from 101 to 200 m^2^ and predominantly in apartments (72.4%). However, a minor proportion of the 31 (10.3%) lived in larger spaces (201 and 400 m^2^).

In addition to the variety in living space sizes, the type of residential setting also varied among respondents. The majority of participants (234, 77.7%) reported living in urban areas during the pandemic, while a smaller portion (63, 20.9%) resided in suburban areas.

When asked to indicate the main sport or sport discipline they practiced, individual sports emerged as the most practiced type of PA among the participants, with a total of 128 (42.5%). Importantly, during the pandemic, the most frequently reported barrier for exercise was the closure of sports facilities and the cancelation of events, which affected more than half of the respondents (52.2%). A considerable number of respondents reported personal and motivational factors (114, 37.9%). A minor proportion 30 (10%) identified fear of COVID-19 infection and health-related concerns as barriers. This reflects the multifaceted challenges faced by individuals in maintaining PA during this period.

### 3.2. Total Weekly Physical Activity Among the Participants

Table 2 presents the findings related to the total PA levels among respondents, which were calculated as the sum of MET-minutes per week from walking, moderate, and vigorous activities. Among the 301 respondents, the majority 200 students (66.4%) achieved high PA levels (>3000 MET-min/week), which is considered sufficient. In contrast, 101 individuals (33.6%) fell into the insufficient category, which includes both moderate (26.6%) and low (7%) activity levels. Although the majority of respondents met the threshold for sufficient PA, the considerable proportion of individuals in the moderate and low categories underscores the need for continued efforts to promote PA, particularly among those not reaching the recommended high levels. The Kruskal-Wallis test was conducted to compare total PA (MET-min/week) among individuals with high, moderate, and low activity levels. The results indicated a statistically significant difference in MET-min/week between these categories (*p* < 0.001).

**Figure 2:**
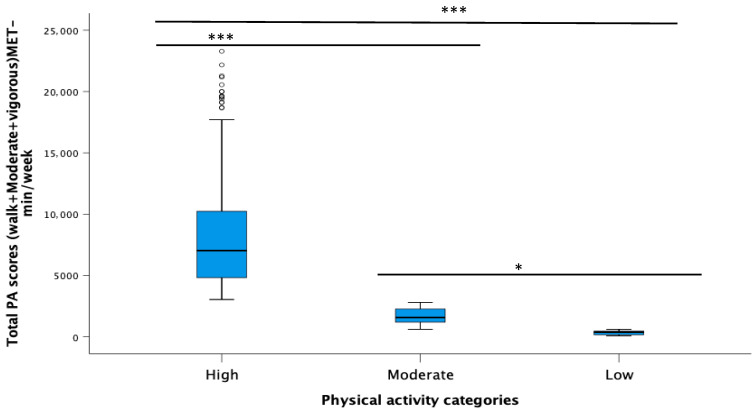
Comparison of total weekly physical activity among low, moderate, and high physical activity categories. * *p* < 0.05, *** *p* < 0.001.

As illustrated in the boxplot (Figure 2), statistically significant differences were observed in the total weekly PA between the low- and High activity groups (*p* < 0.001) as well as between the moderate- and high-activity groups (*p* < 0.001). These findings indicate that individuals in both the low- and moderate-PA groups had significantly lower MET-minutes per week compared to those belonging to High-PA group. Additionally, a significant difference was also observed between the low and moderate groups after applying the Bonferroni correction (*p* = 0.047), suggesting the presence of a graded pattern of PA across all three levels.

**Figure 3:**
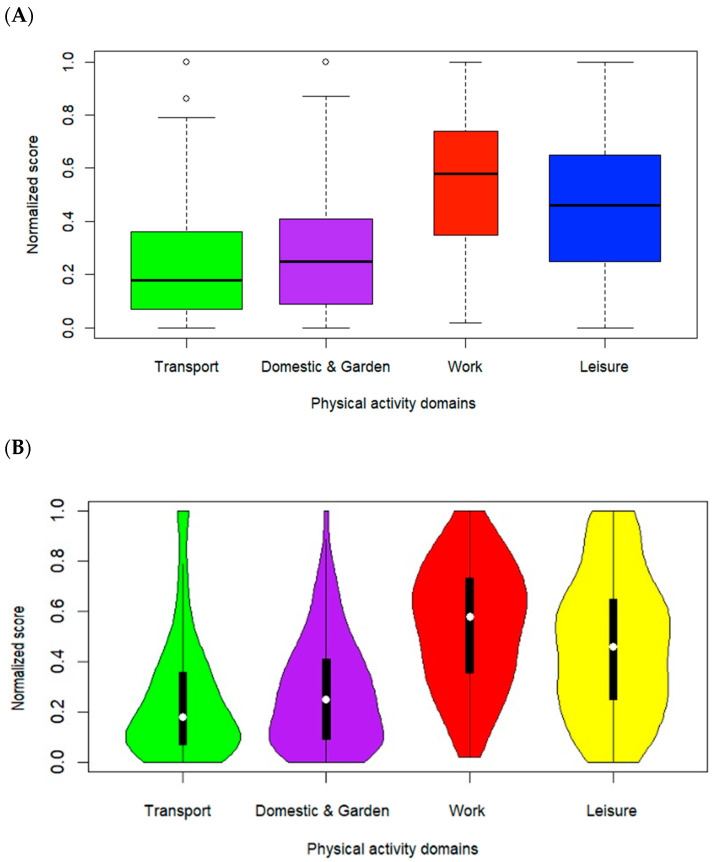
(**A**) Boxplot representing the variability of the normalized ratio of PAs across four IPAQ domains: active transport, domestic and garden, work, and leisure. (**B**) Violin plot displaying the distribution shape and density, highlighting variability and multimodal patterns of PA across the same IPAQ domains within the respondents.

The boxplot shown in Figure 3A provides an overview of the distribution of the PA in the four IPAQ domains (Active Transport, Domestic and Garden, Work, and Leisure), normalized as a proportion of the total PA reported by participants. The median values indicate that the work and leisure domains have the highest levels of PA, whereas the active transport and domestic and garden domains have lower medians. The IQRs suggest high variability in work and leisure activities, whereas active transport and domestic and garden activities exhibit more compact distributions. The boxplots of the active transport, domestic and garden and leisure domains are skewed to the right. However, the work domain is skewed to the left. The violin plot presented in Figure 3B complements the boxplot by providing a more detailed view of the distribution through kernel density estimation (KDE).

The shape of the violin plots indicates that both the work and leisure domains not only have higher median values but also have a wide distribution. This suggests considerable variability, with some individuals engaging in very high levels of PA, whereas others remain relatively inactive. In particular, the leisure domain has a broad and varied distribution, reflecting significant differences in how individuals utilize their leisure time for PA.

In contrast, the Active Transport and Domestic and Garden domains display more concentrated distributions, with fewer individuals engaging in extreme activity levels. The symmetric or asymmetric shapes of the violins help visualize skewness in each category, which is not visible in the boxplot. The violin plot thus offers a more comprehensive representation of PA patterns, making it easier to identify bimodal distributions or varying densities within different domains.

Taken together, the work and leisure domains merge as the primary contributors to overall PA, whereas active transport and domestic activities contribute to a lesser extent.

### 3.3. Gender Differences in Physical Activity Across IPAQ Domains

The gender-based comparison of the normalized PA ratio between the various IPAQ domains is summarized in Table 3. An independent samples *t* test was conducted to compare gender differences across the different domains. In terms of active transport, females (0.2834 ± 0.2639) presented significantly higher levels of PA than males did (0.2172 ± 0.2119), with a mean difference of 0.06622 (*p* = 0.02). In the domestic and garden domains, females (0.3162 ± 0.23503) also had significantly higher activity levels than did male students (0.2332 ± 0.18237), with a mean difference of 0.08305 (*p* = 0.006). For leisure activities, male respondents (0.5317 ± 0.2635) reported significantly higher levels than female respondents did (0.414 ± 0.28089), with a mean difference of −0.11765 (*p* < 0.001). However, no significant difference was found in work-related PA between female (0.5196 ± 0.2599) and male students (0.5789 ± 0.2164), with a mean difference of −0.05922 (*p* = 0.265). These results suggest that gender influences PA patterns across different domains, with females being more active in active transport and domestic activities, whereas males engage more in leisure activities.

### 3.4. Total Physical Activity Stratified by Sociodemographic Characteristics

As shown in Table 4, we compared the means of total PA (expressed in MET-minutes/week) to examine differences across the various sociodemographic characteristics. An independent samples *t* test was used to compare the means of the total PA measured in MET-minutes per week (sum of walking, moderate, and vigorous MET-minutes/week scores) between male and female participants. The results revealed a statistically significant difference in PA between males (7490.77 ± 6697.66) and females (5795.71 ± 5728.44) (*p* = 0.019). Males reported significantly higher total PA levels than females did.

For the other categorical variables, one-way ANOVA was used to compare group means. A significant difference was observed across monthly income groups (*p* = 0.042), with participants earning 500–1000 € reporting the highest PA (8745.21 ± 8058.16), followed by those earning <500 € (6281.18 ± 5484.19) and those earning >1000 € (5889.26 ± 6702.78).

Similarly, significant differences were found across residency types (*p* = 0.001), where participants who were living in rural areas reported substantially greater PAs (17,706.63 ± 17,118.56) than did those living in suburban areas (7085.64 ± 6545.47) or urban areas (6201.00 ± 5682.03). In contrast, no statistically significant mean differences were found by age group (*p* = 0.577) or by residential surface area (*p* = 0.308), indicating that PA levels were relatively similar across these categories.

To examine the effect of employment status on PA levels (MET-min/week). ANOVA revealed a significant difference between the groups (*p* < 0.001). This suggests that employment status influences PA levels. In particular, employed individuals had lower PA levels than other employment categories did. The post hoc analysis shows that unemployed individuals are significantly more physically active than those who are employed part-time or self-employed. However, no significant difference was found between the part-time employed and self-employed groups.

## 4. Discussion

The present study investigated the PA levels among university students at the University of Foggia during the COVID-19 pandemic and identifying the predicting factors of changes in PA pattern. Data were collected using the IPAQ. This period was marked by the emergence of new variants of the SARS-CoV-2 virus, which introduced important disruptions to students’ lifestyle and PA. Although, national restrictions in Italy were gradually eased, persistent health protocols and social distancing have continued to affect students’ daily lifestyles, and limit engagement in various activities. Our collected data provided a valuable insight into the pandemic’s impact on PA and health related behaviors among this Italian students population during a period of global uncertainty, fears and crisis.

The major findings of this study showed that during this pandemic period: (i) The urban residency, low income, and the closure of sports facilities were key barriers to maintain high PA levels among university students. (ii) Despite these challenges, two-thirds of students (66.4%) maintained high levels of total PA. Although, 33.6% of the respondents did not engage in high PA levels, this remains concerning, as insufficient PA poses significant risks to both physical and mental health, particularly during the pandemic period. Significant differences in total PA (MET-minutes/week) were observed across all the PA groups, reflecting stratified impact on student activity levels during the pandemic. (iii) PA level among participants was predominantly concentrated in the Work and Leisure domains, which showed the highest median of the total PA (Met-min/week). In contrast, Active Transport and Domestic and Garden domains contributed less to overall PA and demonstrated more uniform, lower activity levels. These patterns suggest that students relied heavily on work-related and leisure-time activities to maintain optimal level of PA, while other domains played a more limited role. (iv) Gender-based analysis revealed distinct domain-specific differences in PA patterns. Despite, male students showed higher mean of PA levels in the leisure domain, the difference remains not significant between them. Similarly, no significant gender difference was observed in work-related PA. Female students engaged significantly more in active transport and domestic activities compared to male respondents. These findings highlight the influence of gender on domain-specific PA engagement during the pandemic. (v) Taken together, total PA levels varied significantly across key sociodemographic variables including males, mid-income, rural residents, and unemployed students reported higher PA levels, whereas age and living space showed no significant effects. These findings highlight the differential influence of socioeconomic conditions on PA engagement during the COVID-19 pandemic.

Existing literature associates lockdown measures with decreased PA, which may increase risks to both physical and mental health [31]. Survey-based studies have also highlighted the multifactorial nature of these changes, influenced by both individual and contextual restrictions [32]. Moreover, research studies from various countries [25,32,33,34,35,36] consistently report declines in both PA levels and variety during home-confinement period. In contrast, our findings reveal that 66.4% of participants maintained high levels of PA during the pandemic, while 33.6% reported moderate to low PA levels. These results align with recent studies [10,37,38]. This resilience aligns with previous evidence suggesting that students with pre-existing active lifestyles were more likely to maintain their routines, while those previously inactive may have increased their activity levels under restrictive conditions, contributing to the overall higher proportion of high PA among respondents [39,40,41].

In the context of social restrictions and limited access to outdoor activities intensified by anxiety and stress associated with vaccination pass requirements [42]. our study participants sustained their PA mainly through work-related and leisure-time activities. Domestic responsibilities and active transportation contributed to a lesser extent. The elevated PA levels observed in work and leisure domains suggest an adaptive shift in activity patterns during the pandemic. Some students demonstrated resilience by engaging more in flexible or home-based work activities and incorporating recommended forms of leisure-time physical activity [43].

The right-skewed distribution observed in the Leisure and Domestic domains suggests that while the majority of participants maintained moderate activity levels, a subset engaged in significantly higher levels potentially as a coping strategy or as a result of increased time spent at home. This is consistent with findings from European university populations [44].

In contrast, the more centralized distributions and lower levels of activity in Active Transport and Domestic and Garden activities likely reflect the impact of mobility restrictions and the reduced necessity for travel or non-job-related tasks. Furthermore, our data showed considerable heterogeneity in Work and Leisure activity patterns, underlining the differential impact of lockdown on individuals’ capacity and opportunities to remain active in the different PA domains.

These findings mirror the broader literature on the pandemic’s complex influence on PA, showing that while some individuals struggled to maintain sufficient PA, others sustained or even enhanced their PA routines [45,46,47]. Although the pandemic introduced numerous barriers to PA, evidence from various studies indicate that many university students maintained or even increased their PA, particularly through recreational and strength-based activities. These patterns were associated with better psychological outcomes, highlighting their resilience and coping strategies under health crisis conditions [48]. The observed effects are not only influenced by the different stages of the pandemic from lockdown to the easing of restrictions, but also by various factors, including the improved mental health outcomes [49,50].

The analysis of domain-specific PA reveals significant gender differences. Female students reported significantly higher normalized PA levels in active transport and domestic/garden activities compared to their male participants. This is reflecting that both traditional gender roles and adaptive responses to altered daily routines during this period of health crisis [25,51]. Policy changes also played a significant role. The easing of certain restrictions such as the reopening of parks and green spaces was associated with an increased overall average total PA among students. Conversely, the partial reopening of educational institutions, continued public health protocols, and the psychological burden of prolonged isolation may have diminished motivation for regular PA in some individuals.

These gender-based differences are consistent with previous results from Italian studies, which reported that women were more likely to engage in walking and short distance commuting during lockdown, possibly due to reduced access to transportation and an increased focus on health [29,52]. Furthermore, those female students with pre-existing active lifestyles were more likely to maintain adequate PA levels during confinement. Overall, these findings highlight the complex interaction between gender, lifestyle domains, and external restrictions during public health crisis. They underscore the need for tailored strategies to promote PA among the university students, especially in preparation for future disruptions.

The increased domestic activity reported by female participants during the pandemic may reflect a broader tendency for female to take on a greater share of household and caregiving responsibilities, a pattern evident even among student populations [53]. While this form of PA contributes to the overall energy expenditure, it may not provide the same cardiovascular or psychological benefits associated with structured physical activities [54]. In contrast, male students reported significantly higher engagement in leisure-time PA, typically associated with planned, structured, and recreational physical activities such as gym workouts, running, or other sports. Despite the restricted access to sports facilities during the data collection period, many male students maintained their leisure PA routines, aligning with European studies showing greater male resilience in sustaining structured exercise, often attributed to better access to equipment, outdoor space, and performance-oriented motivation [29,55,56,57].

Leisure-time PA also served as a stress-coping strategy during the pandemic, particularly for male students, who were more often relying on exercise to reduce stress and anxiety. As expected, no significant gender differences emerged in work-related PA, likely due to the shift to remote learning and widespread disruption of part-time employment. This transition has led to similar reduction in occupational PA in both male and female students, given the limited physical engagement in campus activities, internships, and service sector jobs [58,59]. These findings are in line with previous studies showing that the pandemic reinforced gender disparities in PA patterns [60]. The total PA varied significantly based on key sociodemographic variables, reflecting the interplay between individual behavior and structural socioeconomic and environmental determinants.

A prominent and consistent finding was the significant gender disparity in PA, with male reporting higher average PA (MET-min/week) than female students. This aligns with the existing evidence indicating higher male participation in moderate-to-vigorous PA, often linked to cultural factors, intrinsic motivation, and the access to sport facilities [29,35,61]. The pandemic appears to have reinforced these disparities, as male participants may have experienced fewer constraints to maintain their PA levels. In contrast, female respondents were more susceptible to increase the sedentary behavior, due to the domestic responsibilities, psychosocial stressors and higher vulnerability to depression [62,63,64].

Numerous studies conducted among European populations show that the lockdown measures temporarily reduced gender disparities in vigorous PA. However, as restrictions eased, there was a return of traditional male-dominant activity patterns. This is largely due to cultural norms, gender differences in sports motivation, access to facilities, and confidence in public exercise settings [65,66,67].

Monthly income has also emerged as a significant predictor of PA. Students in the mid-range income group (500–1000 €) reported the highest PA levels, suggesting a U-shaped relationship between economic status and PA levels. Specifically, lower-income students may lack access to structured PA opportunities, while higher-income students often face time restrictions from academic or professional obligations that reduce their available time for exercise. This is consistent with the findings on socioeconomic disparities in lifestyle behaviors during and after pandemic-related lockdowns [45,68]. Economic flexibility in the mid-income group may have facilitated investment in home-based fitness tools or outdoor activities encouraging PA engagement. The residential context has further influenced PA levels, with rural residents reporting significantly higher activity than suburban or urban peers. This reflects the positive role of the environmental factors, such as access to open space, nature, and lower population density in promoting active lifestyle during unusual restrictive periods. Previous studies demonstrated that urban students were more affected by limitations in space and the closure of recreational facilities [69,70]. Additionally, elevated PA levels among students living in rural areas may include non-leisure PA (e.g., walking long distances, agricultural labor), often underrepresented in urban-focus analyses of student health.

Furthermore, employment status also influenced significantly PA levels, with unemployed students displaying higher activity levels than the employed or self-employed participants. This is likely due to differences in time availability, stress, and routine disruption. Students balancing work or freelance tasks during recovery phases may have deprioritized exercise in favor of financial or academic obligations, as previously documented [65,71]. The absence of a significant difference between the part-time employed and self-employed groups indicates that any form of employment impose limitations on regular PA participation. In contrast, no significant differences were observed based on age or residential surface area. The age homogeneity typical of university cohorts may explain the nonsignificant variation between age groups [72], while surface area may not accurately reflect usable exercise space, especially in shared accommodations or densely constructed urban housing [62]. Together, these findings demonstrate clearly how socioeconomic flexibility, employment, and living conditions significantly influence PA engagement among students, especially during public health crisis. These factors can either amplify or alleviate the impact of restrictive measures on students well-being.

Collectively, these findings underscore that PA engagement among university students during the COVID-19 recovery period was shaped by a multifaceted interplay of social, economic, and physical determinants, rather than being attributed only to individual choice. The combined influence of gender, socioeconomic status, residential context, and employment status emphasizes the need for health promotion strategies informed by an intersectional framework. To address health disparities and promote equitable engagement in PA, post-pandemic public health interventions should incorporate targeted measures, including gender-responsive support systems, subsidized access to fitness resources, and the equitable distribution and accessibility of urban green spaces.

## 5. Study Advantages and Limitations

### 5.1. Advantages

First study to investigate PA patterns among University of Foggia students during the COVID-19 pandemic.

Comprehensive assessment across multiple PA domains: leisure, work-related, domestic, and active transport.

Consideration of gender, socioeconomic, and environmental factors allows for in-depth analysis.

Timely relevance given remote learning, academic stress, and public health challenges.

### 5.2. Study Limitations

The cross-sectional nature of the study prevents conclusions about causality.

Reliance on self-reported PA may introduce recall or reporting bias.

Sample may not fully represent all university students.

Certain determinants of PA (e.g., pre-pandemic habits, mental health status) may not have been fully investigated.

## 6. Study Practical Implications

Develop infrastructure that supports active commuting and leisure-based PA to enhance engagement.

Promote regular PA to improve cognitive function, memory, concentration, and overall academic performance.

Use structured PA programs to reduce academic stress and support adaptive coping strategies during public health emergencies.

Encourage regular PA to enhance mental health, stress regulation, immune function, and overall students physical well-being.

Implement targeted interventions to increase leisure-time PA among female students and other at-risk groups to reduce health disparities.

Foster sustainable healthy behavior that promotes resilience during and beyond crises.

## 7. Conclusions

During the COVID-19 pandemic, Students at University of Foggia demonstrated notable resilience by maintaining relatively high levels of PA, mainly through work-related and leisure activities, despite various obstacles. However, one-third of the interviewed population remained insufficiently active, indicating that elevated overall PA did not uniformly translate into adequate engagement across all domains.

PA patterns varied significantly by gender, income, residence, and employment status, underscoring the complex interplay of socioeconomic and environmental determinants influencing student behavior during a global health crisis. Male students reported higher levels of leisure-time PA, while females engaged more in domestic and active transport-related activities. This underscores the resilience and adaptive behaviors that supported both physical and mental health well-being. These gender-specific differences emphasize the importance of adopting a domain-specific lens when assessing PA and designing PA interventions in post-pandemic university settings. However, reliance on non-leisure domains particularly domestic activities among female students raises concerns regarding autonomy, enjoyment, and long-term sustainability of PA and behaviors. Policies that support active commuting infrastructure, gender-sensitive leisure programs, and equitable domestic labor distribution could help address disparities. Health promotion efforts should therefore prioritize equitable, accessible and gender-sensitive opportunities that encourage voluntary engagement in leisure-time PA rather that dependence n obligatory domestic roles to meet activity thresholds.

Given the well-documented protective role of regular physical activity in supporting mental health, stress regulation, immune function and overall well-being during periods of social and psychological disruptions, universities have a critical responsibility to foster supportive environments for active lifestyles.

For the students population, appropriate engagement in PA is critical to enhance cognitive function, improved memory, better concentration, and enhancing the overall academic performance that are especially vital in the context of remote learning and increased academic stress during the pandemic Integrating flexible, inclusive, and equity-oriented PA initiatives into university policies and curricula may significantly improve student well-being, academic outcomes, and resilience during and beyond public health emergencies, thereby strengthening institutional preparedness for future crises.

## 8. Future Directions

This study underscores the urgent need for universities to implement proactive, evidence-based strategies that support student PA and overall well-being during health crises. By identifying which student groups are most vulnerable and which activity patterns are most resilient, the findings provide actionable guidance for designing targeted interventions that promote both physical and mental health. Future efforts should address PA disparities through an intersectional lens, emphasizing gender-based support, equitable access to affordable fitness opportunities, and expanded green spaces. Promoting virtual and home-based exercise, alongside continued research on pandemic-related health impacts, is essential. Universities can further support students by providing online resources, accessible outdoor options, mental health services, and by integrating PA into higher education curricula. Post-pandemic recovery should prioritize re-engaging students through coordinated policies that sustain well-being and resilience. Collectively, these findings offer higher education institutions a roadmap to safeguard student health, maintain engagement, and enhance adaptability in the face of future disruptions. By translating this evidence into practical programs and policies, universities can not only mitigate the negative impacts of crises but also cultivate a lasting culture of health and well-being that supports students’ long-term cognitive abilities and learning outcomes in higher education institutions.

## Figures and Tables

**Table 1:** Demographic characteristics of the study population.

Variables	Characteristics	Frequency (N)	Percentage (%)	Mean ± SD
Age categories	18–20	148	49.2	22.03 ± 4.9
	21–30	136	45.2	
	31 and above	17	5.6	
Gender				
	Male	132	43.9	
	Female	169	56.1	
Educational level				
	Undergraduate	272	90.4	
	Graduate/Postgraduate	17	5.6	
	Other	12	4	
Living status				
	Living with families/others	285	94.7	
	Living alone	16	5.3	
Monthly income				
	<500 €	193	64.1	
	500–1000 €	42	14	
	>1000 €	66	21.9	
BMI (kg/m^2^)				22.42 ± 3.77
	Underweight (<18.5)	28	9.3	
	Normal weight (18.5–24.9)	222	73.8	
	Preobese (25–29.9)	46	15.3	
	Obese (Class I, II and III)	5	1.7	
Indicate the number in square meters (m^2^) in which you live in this period (COVID-19 pandemic):				
	10–100	145	48.2	
	101–200	125	41.5	
	201–400	31	10.3	
Indicate the type of house where you live in this period (COVID-19 pandemic):				
	Urban residency	234	77.7	
	Suburban residency	63	20.9	
	Rural residency	4	1.3	
Which sport/sport discipline do you practice? (Indicate only the main sport)				
	Individual Sports	128	42.5	
	Collective sports	67	22.3	
	No main sports/other	106	35.2	
Reported exercise obstacles during the COVID-19 pandemic				
	Infrastructure closure and canceled sports events	157	52.2	
	Fear of COVID-19 and Health- Related Concerns	30	10	
	Personal and Motivational Factors	114	37.9	
Employment status				
	Employed (part-time)	36	12	
	Self-employed	24	8	
	Unemployed	241	80.1	

**Table 2:** Total weekly Physical Activity Levels and Their Prevalence Based on the IPAQ Scoring Protocol.

Total PA (Walk + Moderate + Vigorous)	Frequency (N)	Percentage (%)	*p* Value
High (>3000 MET-min/week)	200	66.4	
Moderate (600–3000 MET-min/week)	80	26.6	<0.001
Low (<600 MET-min/week)	21	7	
Total number of respondents	301	100	

**Table 3:** Gender-based comparison of the normalized PA ratio across the four IPAQ domains.

IPAQ Domains	Gender	N	Mean ± SD	Levene’s Test (F, *p* Value)	t (df)	*p* Value	MD	95% CI (Lower, Upper)
Active Transport	Female	151	0.28 ± 0.26	6.122 (0.014)	2.30 (272)	0.022	0.07	(0.00962, 0.12283)
	Male	123	0.22 ± 0.21					
Domestic and Garden	Female	111	0.32 ± 0.24	7.261 (0.008)	2.76 (191)	0.006	0.08	(0.02376, 0.14233)
	Male	82	0.23 ± 0.18					
Work	Female	55	0.52 ± 0.26	1.362 (0.246)	−1.12 (88)	0.265	−0.06	(−0.16407, 0.04563)
	Male	35	0.58 ± 0.22					
Leisure	Female	151	0.41 ± 0.28	0.624 (0.43)	−3.55 (273)	0.000	−0.12	(−0.18283, −0.05247)
	Male	124	0.53 ± 0.26					

MD: Mean difference; N: Frequency.

**Table 4:** Total physical activity (MET-min/week), stratified by sociodemographic characteristics.

		Group	N	Mean ± SD (MET-Min/Week)	*p* Value	Levene’s Test (F, *p* Value)
Total Physical activity (MET-min/Week)	Gender	Female	169	5795.71 ± 5728.44	0.019	
		Male	132	7490.77 ± 6697.66		0.092 (0.762) (*t* test)
	Monthly Income	<500 €	193	6281.18 ± 5484.19	0.042	
		500–1000 €	42	8745.21 ± 8058.16		
		>1000 €	66	5889.26 ± 6702.78		3.216 (ANOVA)
	Age Group	18–20	148	6912.48 ± 6289.51	0.577	
		21–30	136	6136.07 ± 6118.75		
		31–45	17	6512.07 ± 6568.45		0.551 (ANOVA)
	Surface Area (m^2^)	10–100	145	6179.95 ± 5924.51	0.308	
		101–200	125	6576.77 ± 6366.58		
		201–400	31	8066.68 ± 6905.58		1.181 (ANOVA)
	Residency Type	Urban	234	6201.00 ± 5682.03	0.001	
		Suburban	63	7085.64 ± 6545.47		
		Rural	4	17,706.63 ± 17,118.56		7.336 (ANOVA)
	Employment status	Employed (part-time)	36	10,229.05 ± 9132.27	0.000	
		Self-employed	24	10,786.69 ± 6884.25		
		Unemployed	241	5564.85 ± 5180.66		16.42 (ANOVA)

## Data Availability

The data presented in this study are available on request from the corresponding author, due to privacy and ethical considerations related to the anonymity of the human participants, the data cannot be made publicly available.
